# Validation of serum progesterone <35nmol/L as a predictor of miscarriage among women with threatened miscarriage

**DOI:** 10.1186/s12884-017-1261-4

**Published:** 2017-03-06

**Authors:** Sze Min Lek, Chee Wai Ku, John C. Allen Jr, Rahul Malhotra, Nguan Soon Tan, Truls Østbye, Thiam Chye Tan

**Affiliations:** 1 Duke-National University of Singapore Medical School, 8 College Road, Singapore, 169857 Singapore; 2 School of Biological Sciences, Nanyang Technological University, 50 Nanyang Ave, Singapore, 639798 Singapore; 3 Institute of Molecular and Cell Biology, A*STAR, 61 Biopolis Drive, Proteos, Singapore, 138673 Singapore; 4 Department of Obstetrics and Gynecology, KK Women’s and Children’s Hospital, 100 Bukit Timah Road, Singapore, 229899 Singapore

**Keywords:** Progesterone, Cut-off level, Predictive, Miscarriage

## Abstract

**Background:**

Our recent paper, based on a pilot cohort of 119 women, showed that serum progesterone <35 nmol/L was prognostic of spontaneous miscarriage by 16 weeks in women with threatened miscarriage in early pregnancy. Using a larger cohort of women from the same setting (validation cohort), we aim to assess the validity of serum progesterone <35 nmol/L with the outcome of spontaneous miscarriage by 16 weeks.

**Methods:**

In a prospective cohort study, 360 pregnant women presenting with threatened miscarriage between gestation weeks 6–10 at a tertiary hospital emergency unit for women in Singapore were recruited for this study. The main outcome measure measured is spontaneous miscarriage prior to week 16 of gestation. Area under the ROC curve (AUC) and test characteristics (sensitivity, specificity, positive and negative predictive value) at a serum progesterone cutpoint of <35 nmol/L for predicting high and low risk of spontaneous miscarriage by 16 weeks were compared between the Pilot and Validation cohorts.

**Results:**

Test characteristics and AUC values using serum progesterone <35 nmol/L in the validation cohort were not significantly different from those in the Pilot cohort, demonstrating excellent accuracy and reproducibility of the proposed serum progesterone cut-off level.

**Conclusions:**

The cut-off value for serum progesterone (35 nmol/L) demonstrated clinical relevance and allow clinicians to stratify patients into high and low risk groups for spontaneous miscarriage.

## Background

Threatened miscarriage—defined as an ongoing pregnancy associated with vaginal bleeding, with or without abdominal pain [1]—is the most common gynecological emergency, occurring in 15–20% of ongoing pregnancies [2], with 25% progressing to spontaneous miscarriage [3, 4]. Many clinical resources are utilized in attempting a definitive diagnosis of spontaneous miscarriage among women with threatened miscarriage, as not all are at equal risk of miscarriage. Individualizing outpatient management based on an initial risk evaluation would be a boon to clinical care and suggest any additional therapy.

While maternal factors like older age (>33 years) and lower body mass index (BMI) <20 kg/m^2^ have been found to contribute to spontaneous miscarriage [5], recent focus has been on identifying serum biological markers prognostic of spontaneous miscarriage. A recent meta-analysis by Pillai et al of prospective studies investigating biomarkers to determine pregnancy outcome for women presenting with threatened miscarriage have showed conflicting results with the need for larger studies and further validation [6]. Other studies have looked at various biomarkers such as serum beta HCG, estradiol, PAPP-A, inhibin, CA 125 as well as progesterone. In addition, other studies have also looked at using different combination of maternal demographics, serum biomarkers and ultrasound features to predict pregnancy viability that shows promise but require the implementation of a mathematical model and algorithm. This could prove to be unwieldy in a busy clinical service due to multiple variables that need to be collected in order to use the model effectively [7–10].

Presently, the most promising is serum progesterone. A recent prospective cohort study of women with no signs of threatened miscarriage from 4 to 12 weeks of gestation reported risk of miscarriage to be significantly higher among women with low serum progesterone (<38.3 nmol/L or 12 ng/ml) [5]. Progesterone levels were 48% lower in women experiencing threatened miscarriage with subsequent spontaneous miscarriage compared to women who delivered at term. [9, 11] In our group’s paper, serum progesterone levels were significantly lower in women presenting with threatened miscarriage who subsequently experienced spontaneous miscarriage by 16 weeks gestation compared to those who did not miscarry. A cut-off serum progesterone level of 35 nmol/L was proposed to prognosticate low and high risk for spontaneous miscarriage after presenting with threatened miscarriage in early pregnancy [12]. Notwithstanding these findings, serum progesterone is currently not used for miscarriage risk assessment in a clinical setting. One explanation may be insufficient evidence regarding appropriate or ‘optimal’ serum progesterone cut-off levels for risk stratification of spontaneous miscarriage. Thus, a validated serum progesterone cut-off as a prognostic risk assessment will allow clinicians the means to better stratify patients into low- and high-risk populations.

The current study employed a large, prospective cohort (Validation cohort) with the aim of validating the serum progesterone cut-off value of 35 nmol/L based on the results of an earlier, smaller study at the same institution (Pilot cohort). We compared areas under ROC curves (AUCs) as well as sensitivity, specificity, positive and negative predictive values between the Pilot and Validation cohorts using 35 nmol/L as the risk prognosis cut-off for spontaneous miscarriage at or before 16 weeks of gestation in women presenting with threatened miscarriage in weeks 6–10 of pregnancy.

## Methods

**Fig. 1:**
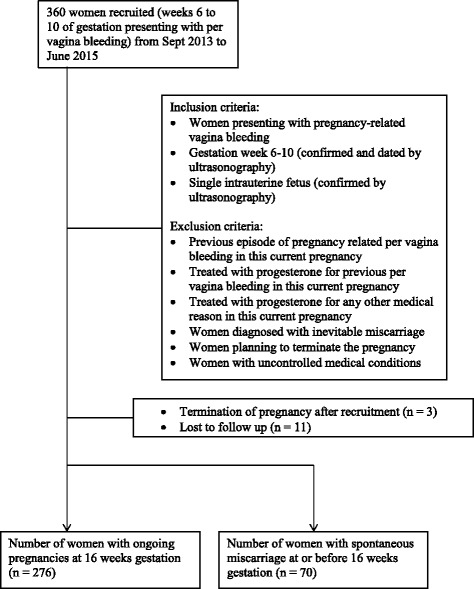
Sample selection flow chart

A total of 360 pregnant women, aged 21 years and above, presenting at the KK Women’s and Children’s Hospital (KKH) 24-h Women’s Clinic from September 2013 to June 2015 were recruited in the Validation cohort. Inclusion criteria were a single intrauterine pregnancy between gestation weeks 6 to 10 (confirmed and dated by ultrasonography), with pregnancy-related per vagina bleeding. Women with previous episodes of per vagina bleeding or those treated with progesterone for previous per vagina bleeding in the current pregnancy, or women diagnosed with inevitable miscarriage, missed miscarriage, blighted ovum or planned termination of pregnancy were excluded (Fig. 1).

Maternal blood samples were taken to measure serum progesterone level at presentation. Blood was collected in plain tubes and centrifuged for 10 min at 3000 *g* within 2 h of collection. Serum progesterone level was measured in the KKH clinical laboratory using a commercial ARCHITECT progesterone kit (Abbott, Ireland).

Covariates for the analysis were maternal demographics, health, obstetric and lifestyle factors collected by an investigator administered questionnaire in either English or Chinese (Table 1).

**Table 1:** Comparison of serum progesterone levels and maternal characteristics at baseline by pregnancy status at 16 weeks gestation for Pilot and Validation cohorts

Maternal characteristics	Ongoing pregnancy at 16 weeks gestation			Spontaneous miscarriage at or before 16 weeks gestation		
	Pilot Cohort (*N* = 89)	Validation Cohort (*N* = 276)	*p* value	Pilot Cohort (*N* = 30)	Validation Cohort (*N* = 70)	*p* value
Serum biological markers						
Progesterone, mean ± SD (nmol/L)	59.8 ± 23.5	60.3 ± 22.7	0.848	26.4 ± 17.1	31.4 ± 20.1	0.237
Demographics						
Age, mean ± SD (years)	29.3 ± 4.83	30.3 ± 4.10	0.0769	31.3 ± 4.73	31.6 ± 5.22	0.774
Age of spouse, mean ± SD (years)	32.9 ± 5.43	33.1 ± 5.96	0.772	33.9 ± 5.10	34.6 ± 6.38	0.628
Race						
Chinese (%)	53.9	52.0	0.271	53.3	38.6	0.396
Malay (%)	19.1	25.5		16.7	31.4	
Indian (%)	16.9	10.2		16.7	14.3	
Others (%)	10.1	12.4		13.3	15.7	
Marital status						
Married	92.1	94.6	0.444	90.0	97.1	0.158
Single	7.87	5.45		10.0	2.86	
Highest educational level						
University degree (%)	42.7	39.3	0.179	46.7	42.9	0.724
ITE or polytechnic (%)	30.3	40.4		36.7	32.9	
Primary and secondary (%)	27.0	20.4		16.7	24.3	
Health, obstetric and lifestyle factors						
Planned Pregnancy (%)	59.6	58.2	0.902	63.3	44.3	0.126
Gestation age at recruitment, mean (wks)	7.49 ± 1.43	7.48 ± 1.47	0.968	6.89 ± 1.30	6.63 ± 1.06	0.311
Fetal pole present (%)	93.3	100	**0.0002**	50.0	100	**<0.0001**
Fetal heart present (%)	87.6	95.6	**0.011**	30.0	70.0	**0.0003**
Number of children						
None (%)	55.1	45.5	0.143	43.3	32.9	0.367
1 or more (%)	44.9	54.6		56.7	67.1	
Previous miscarriage (%)	20.2	24.0	0.563	20.0	22.9	1.000
BMI, mean ± SD (kg/m^2^)	23.7 ± 5.09	22.9 ± 4.29	0.204	22.1 ± 3.81	23.6 ± 4.81	0.0903
Medical comorbidities						
Diabetes mellitus (%)	0	0.360	1.000	0	0	1.000
Hypertension (%)	0	0	1.000	0	0	1.000
Thyroid disease (%)	1.12	1.09	1.000	0	1.43	1.000
Gynecological disease (%)	12.4	12.0	1.000	0	10	0.0991
Smoking during pregnancy (%)	3.37	5.45	0.579	0	5.71	0.313
Exposed second hand smoke at home (%)	40.5	44.7	0.539	30	38.6	0.498
Alcohol during pregnancy (%)	1.12	0.72	1.000	0	0	1.000
Nausea during pregnancy (%)	70.8	77.5	0.203	40	55.7	0.192

Entries in boldface were used to show that these variables were significantly related to different or associated with spontaneous miscarriage

*SD* standard deviation, *CRL* crown-rump length, *BMI* maternal body mass index

### Outcome measures and follow-up

The primary outcome measured was spontaneous miscarriage, defined by self-reported uterine evacuation after inevitable or incomplete miscarriage, or complete miscarriage with an empty uterus, by the 16th week of gestation. All participants were contacted at the 16th week of pregnancy to verify pregnancy status.

### Statistical Methods

The progesterone cut-off of <35 nmol/L for high risk of miscarriage before the 16th week of pregnancy was determined and developed using the Pilot study cohort as reported in Ku et al. [12] The <35 nmol/L serum progesterone cut-off form the basis for our validation study.

Baseline maternal demographics and pregnancy characteristics were statistically compared between the Pilot and the Validation cohorts with respect to two patient subgroups: (i) patients who experienced spontaneous miscarriage at 16 weeks of gestation and (ii) patients with ongoing pregnancy at 16 weeks gestation. The 2-sample *t*-test was used to compare continuous baseline variables and Fisher’s exact test to compare categorical variables.

Logistic regression equations were fitted to the respective Pilot and Validation cohort data sets. The outcome was the binary response ‘Yes/No’ corresponding to occurrence/non-occurrence of spontaneous miscarriage. The single predictor was progesterone level (nmol/L). Parameter estimates were obtained for the intercept (*b*_0_) and progesterone coefficient (*b*_1_). ROC analysis was performed using progesterone concentration as a continuous variable. The Youden criterion, which consists of identifying the value of progesterone that maximizes the sum of sensitivity and specificity, was applied to identify the ‘optimum’ progesterone cut-off for each cohort. ROC curves and AUCs for the Pilot and Validation cohorts were computed and compared statistically the using the approach of Hanley and McNeil for comparing independent ROC curves derived from different samples. 95% confidence intervals on the difference between the Pilot and Validation AUCs were obtained.

Using the progesterone cut-off of <35 nmol/L, test parameters of sensitivity, specificity, PPV and NPV were estimated and compared between the Pilot and Validation cohorts using Fisher’s exact test. Exact Clopper-Pearson 95% confidence intervals were calculated on test parameter estimates. Differences in Pilot and Validation parameter estimates were obtained and 95% confidence intervals on differences were calculated using a normal approximation approach. Likelihood ratios corresponding to positive (LR+) and negative (LR-) test outcomes were calculated. Agreement of actual versus predicted Validation cohort miscarriages based on the <35 nmol/L cut-off was assessed using McNemar’s test. Corresponding confidence intervals on the difference were calculated using the Wald *z* method for differences of correlated proportions. Analyses were performed using SAS software version 9.3 (SAS Institute Inc., Cary, North Carolina, USA).

## Results

Of the 360 women recruited in the Validation cohort, 11 were lost to follow up and 3 withdrew from the study due to induced termination of pregnancy. Of the 346 women included in the analysis, 70 (20.2%) experienced spontaneous miscarriage prior to week 16. Of the 119 women in the Pilot cohort, 30 (25.2%) experienced spontaneous miscarriage prior to week 16 of gestation. The difference (95% Confidence Interval (CI)) in incidence proportions between the Pilot and Validation cohorts was 0.05 (-0.03, 0.14) and not statistically significant (*p* = 0.301).

Serum progesterone levels (nmol/L) did not differ significantly between Pilot (P) and Validation (V) cohorts for women with ongoing pregnancies at 16 weeks (P-59.8, V-60.3; *p* = 0.848) or women experiencing spontaneous miscarriage prior to week 16 (P-26.4, V-31.4; *p* = 0.237). In women with ongoing pregnancy at week 16, the only maternal demographic and clinical variables exhibiting a statistically significant difference between the two cohorts were presence of fetal pole (%) (P-93.3, V-100; *p* = 0.0002) and fetal heart (%) (P-87.6, V-95.6; *p* = 0.011). The same held true in women with spontaneous miscarriage prior to week 16, with significant differences in presence of fetal pole (P-50.0, V-100; *p* <0.0001) and fetal heart (P-30.0, V-70.0; *p* = 0.0003) (Table 1).

Logistic regression equations parameter estimates for the intercept (*b*_0_) and progesterone coefficient (*b*_1_) were (*b*_0_, *b*_1_) = (2.8439, -0.0971) for the Pilot cohort and (2.1063, -0.0782) for the Validation cohort. Receiver Operating Characteristic (ROC) analysis was performed on the Validation cohort data with progesterone level as a continuous variable.

### Validation of serum progesterone for prognosticating risk of spontaneous miscarriage

**Fig. 2:**
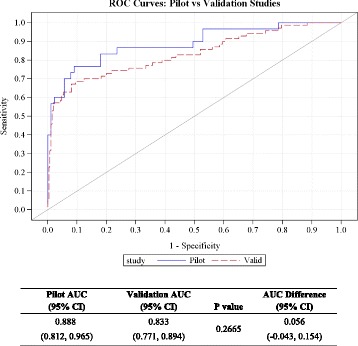
Comparison of ROC curves for serum progesterone from Pilot and Validation cohorts at < 35 nmol/L cut-off

AUC (95% CI) for the Pilot and Validation cohort serum progesterone ROC curves was 0.89 (0.81, 0.97) and 0.83 (0.77, 9.89), respectively. The difference (95% CI) was 0.06 (-0.04, 0.15) and not statistically significant (*p* = 0.267) (Fig. 2). In applying the Youden index criterion to the ROC curve, the progesterone concentration in the Validation cohort that maximized sensitivity + specificity was 35 nmol/L, which was consistent with the Pilot cohort result. For the Validation cohort (<35 nmol/L cut-off), test sensitivity = 65.7%, specificity = 92.0%, Positive Predictive Value (PPV) = 67.7%, Negative Predictive Value (NPV) = 91.3%, LR + = 8.21 and LR- = 0.37 for spontaneous miscarriage by week 16. There were no significant differences between the Pilot and Validation cohorts among any test performance parameters (Table 2).

**Table 2:** Comparison of sensitivity, specificity, PPV, and NPV between Pilot and Validation cohorts for progesterone at cut-off < 35 nmol/L for high risk of miscarriage before 16th week of pregnancy

Statistic (95% CI) Counts	Study		*P*-value^1^	Diff (95% CI)
	Pilot	Valid		
Sensitivity	0.767 (0.577, 0.901) 23/30	0. 657 (0.534, 0.767) 46/70	0.349	0.110 (-0.093, 0.274)
Specificity	0.888 (0.803, 0.945) 79/89	0. 920 (0.881, 0.949) 253/275	0.389	-0.032 (-0.119, 0.031)
PPV	0.697 ^a^ (0.513, 0.844) 23/33	0. 677 ^b^ (0.552, 0.785) 46/68	1.000	0.021 (-0.177, 0.196)
NPV	0. 919 ^a^ (0.839, 0.967) 79/86	0. 913 ^b^ (0.874, 0.944) 253/277	1.000	0.005 (-0.077, 0.062)
LR+	6.82	8.21		
LR-	0.26	0.37		

^1^ Fisher’s exact test, ^a^ Prevalence = 0.25; ^b^ Prevalence = 0.20

### Agreement of actual versus predicted miscarriages by 16 weeks in the validation cohort

Using the <35 nmol/L serum progesterone cut-off, among the 346 women in the validation cohort, results were True Positive = 46, True Negative = 254, False Positive = 24 and False Negative = 22 translating to accuracy (95% CI) of 300/346 = 0.87 (0.83, 0.90). The null hypothesis of equality of the proportion of miscarriages predicted by the model (0.197 = 68/346) versus the actual proportion (0.202 = 70/346) was non-significant by McNemar’s test (*p* = 0.768) for the difference (95% CI) of -0.006 (-0.044, 0.033).

## Discussion

There is significant interest in developing clinically useful models for risk prognosis of pregnancy complications and outcomes, and to stratify pregnant women as low or high risk. The advantages of serum progesterone as a marker for spontaneous miscarriage in women with threatened miscarriage are three-fold: (1) high reliability for reassuring women at low risk of miscarriage (NPV > 90%), (2) provides clinical guidance for unnecessary progestogen treatments or bed rest, and (3) may prompt mobilization of resources supporting an expectant mother’s psychological wellbeing.

### Main findings

Our study was targeted at women presenting with threatened miscarriage in early pregnancy in order to validate a previously suggested serum progesterone cut-off level of <35 nmol/L for predicting risk of spontaneous miscarriage in this high-risk population. In the past, a reliable marker or model for clinical prognosis of pregnancy outcome in women presenting with threatened miscarriage in early pregnancy has not been available. In this study, we have successfully validated the serum progesterone cut-off value of <35 nmol/L (11 ng/ml)—originally identified in a previous study [12], as a clinically useful predictor of miscarriage prior to week 16 of pregnancy in a temporally different population from the same centre. Using this cut-off, individual patients can be quickly stratified as being at low risk or high risk of spontaneous miscarriage.

### Strengths and interpretation

Early pregnancy is maintained via mediation by hormones and endocrine-immune interactions [13]. Progesterone is increasingly recognized as a critical hormone during implantation where it plays an important role in sustaining decidualization, controlling uterine contractility and promoting maternal immune tolerance to the fetal semi-allograft. [14] Thus, high level of serum progesterone may be protective against early pregnancy loss. In contrast, low level of serum progesterone could contribute to an increased risk of subsequent spontaneous miscarriage, especially in women who presents with threatened miscarriage in the first trimester of pregnancy. However, very few studies have reported a specific cut-off progesterone level with high predictive value for spontaneous miscarriage [11, 15–17].

The serum progesterone cutoff level of <35 nmol/L originally proposed by Ku et al [12] provides important information that can be used to provide individualized and patient-specific risk assessment for spontaneous miscarriage. This provides prognostic data available at entry to care. Our validated proposed cut off level for serum progesterone allows clinicians to quickly assess individual patient risk by calculating a probability based on presence or absence of various factors. This threshold level for serum progesterone can be applied in a clinical setting, accurately identifying high-risk patients who may require more therapeutic intervention or heightened surveillance. In addition, this subpopulation of patients may potentially contribute to further pharmacological intervention studies whereby its therapeutic effectiveness and efficacy can be determined. Although measurements of serum progesterone levels are not recommended for routine clinical use at this time, it can be determined that serum progesterone is a highly specific predictive biomarker for spontaneous miscarriage, with a high negative predictive value, allowing us to reassure anxious patients with a low risk of miscarriage. Finally, we are able to demonstrate that our serum progesterone cut off value of 35 nmol/L was highly reproducible in a temporally different patient population, thus supporting the validity of the model.

### Limitation

However, our study has a few limitations. This proposed serum progesterone cut-off value was shown to be applicable in women who present with threatened miscarriage in early pregnancy. It is uncertain if the predictive values will be applicable in a low-risk population, but the sensitivity and the specificity should remain constant, as these parameters are independent of disease prevalence. External validation is required before widespread implementation into clinical practice is possible.

## Conclusion

We present a proposed cut off serum progesterone value of <35 nmol/L as a validated threshold level to predict spontaneous miscarriage, which demonstrates both excellent accuracy and acceptable reproducibility. This threshold level allows clinicians to perform risk stratification relating to risk of spontaneous miscarriage, allowing for better prognostication and guiding therapeutic interventions. This model is both clinically applicable and easily implemented, as well as provides research opportunities for future miscarriage intervention studies.

## Abbreviations

AUC: Area under the ROC curve (AUC)
BMI: Body mass index
CI: Confidence interval
KKH: KK women’s and children’s hospital
LR: Likelihood ratio
NPV: Negative predictive value
P: Pilot
PPV: Positive predictive value
ROC: Receiver operating characteristic
V: Validation
